# Coalition building and citizen science for radon risk reduction

**DOI:** 10.1088/2752-5309/ae4e50

**Published:** 2026-03-20

**Authors:** Stacy R Stanifer, Kathy Rademacher, Whitney Sedio, Naomi Cheek, David Gross, Caitlyn Curtis, Amanda Thaxton-Wiggins, Mary Kay Rayens, Ellen J Hahn

**Affiliations:** 1 University of Kentucky, College of Nursing, 751 Rose Street, Lexington, KY 40536, United States of America; 2 Northeast Kentucky Area Health Education Center, 316 W. Second Street, Morehead, KY 40351, United States of America; 3 University of Kentucky, College of Public Health, 111 Washington Ave, Lexington, KY 40536, United States of America

**Keywords:** radon, rural population, citizen science, coalition

## Abstract

We evaluated the implementation of local coalitions led in partnership with citizen scientists, community-based organizations, and public libraries in four rural communities to lower exposure to radon in the home. The objectives were to (1) describe the Reach-Effectiveness-Adoption-Implementation-Maintenance (RE-AIM) of four radon coalitions, and (2) compare RE-AIM factors among citizen scientists who participated in the coalitions and those who did not. A larger community-engaged research project embedded coalition building using a citizen science approach. Three of the four coalitions focused on health and wellness more broadly (18–34 members); one focused solely on radon (10 members). Coalition membership and activities varied from marketing a radon detector Library Loan Program, community events, and in-library tabling events to working with government officials to sign National Radon Action Month proclamations. We used mixed methods to evaluate coalition-building using the RE-AIM framework. The coalitions were most likely to reach local health departments, hospitals, and schools. Although these partners were highly supportive, they provided few to no resources. Four in 10 citizen scientists were at least moderately involved in the coalition regardless of whether they had high home radon. Citizen scientists reported low awareness of both how frequently radon received local media attention and how favorably radon awareness, testing, and mitigation was portrayed in local media, particularly among those uninvolved in the coalition. Citizen scientists involved in the coalition had the most experience disseminating scientific information on radon and educating the public. The coalitions fostered radon mitigation as 82% of library loan participants with high radon were likely to hire a radon mitigation professional, and all said financial assistance would help them mitigate. Multi-issue health coalitions that engage citizen scientists and partner with public libraries can increase radon testing and build demand for mitigation in rural areas.

## 1. Introduction

A coalition is typically defined as a group of people from diverse organizations or community sectors who agree to tackle a common goal. Systematic evaluation to determine coalition effectiveness in addressing public health challenges is essential to establish whether efforts translate into meaningful change and guide coalition-based interventions. However, there is little research that investigates the structures or processes of coalitions nor are there many studies that test the effectiveness of community coalitions [1].

Coalitions of like-minded individuals often form to tackle various persistent public health challenges such as obesity [2, 3], as well as to foster environmental justice [4], and build resilience in the face of a pandemic like COVID-19 [5]. Others promote wellness in the community more broadly and are sometimes led or facilitated by Cooperative Extensions [2, 3, 5, 6] and libraries [7, 8]. Radon, a ubiquitous environmental carcinogen, is a global public health challenge. In the U.S., radon exposure is responsible for approximately 21 000 lung cancer deaths annually [9], and worldwide between 0.2% and 26% of lung cancer cases are attributed to radon exposure [10–12].

Radon coalition examples include one cross-organization health coalition with eight stakeholders in Montana formed to promote home radon testing by combining tobacco cessation with radon screening [13]. In other states such as Iowa, statewide radon coalitions work in collaboration with public health and cancer groups to increase radon testing and mitigation, and promote radon policy change [14]. Despite the wide-spread risk of radon exposure, only two-thirds of the 65 National Comprehensive Cancer Control Program coalitions in the U.S. focused on radon as of 2015, although there has been some progress in more recent years [15, 16]. Many of these cancer coalitions have had success advocating for state radon laws, mandating disclosure of radon in real estate transactions and licensing radon professionals [16].

Methods of coalition evaluation vary [17] and often assess whether objectives are met, are used to improve program implementation, inform policy decisions, and provide accountability to funders, to name a few. Researchers often use the Reach-Effectiveness-Adoption-Implementation-Maintenance (RE-AIM) framework [18] to evaluate health programs and policies. For example, Kupershmidt *et al* used RE-AIM to evaluate the key features and obstacles to implementing a statewide coalition of Advance Care Planning educators [19]. Additionally, Knudsen *et al* adapted the RE-AIM framework to evaluate implementation fidelity and context using repeated annual surveys and qualitative interviews with coalition members and stakeholders in the Communities that HEAL intervention to reduce opioid overdose deaths [20]. While few researchers employ qualitative approaches when using the RE-AIM framework, mixed methods including qualitative methods can provide a richer understanding of complex situations like coalition-building [21].

## 2. Purpose and objectives

The purpose of this paper is to evaluate the development and implementation of four local coalitions led in partnership with citizen scientists, community-based organizations, and public libraries in four rural Kentucky, U.S. communities to lower exposure to radon in the home. As part of a larger study, Residents Acting to Detect and Alleviate Radon (RADAR; 2020–2024), rural residents were recruited and trained as citizen scientists [22] to test their homes for radon and promote a radon detector public Library Loan Program (LLP) [23, 24]. The four rural counties were purposively selected based on their radon risk potential and then matched on county-level median income and population size. In each of the four rural study counties, citizen scientists, area health education centers, public libraries, and economic development organizations worked in partnership to create, grow, maintain, and sustain, or build on existing coalitions.

The objectives of this paper are to (1) describe the RE-AIM of four radon coalitions, and (2) compare RE-AIM factors among citizen scientists who participated in the coalitions and those who did not. A total of 60 citizen scientists (20 per study county) were initially recruited to the RADAR project. The citizen scientists were educated on how to use the digital radon detector (i.e. Corentium Home by Airthings®) to test their homes, and they advised the research team on the usability of the detector, how to report back the data, and plan for community action [22]. Some citizen scientists were more engaged in coalition activities than others.

## 3. Intervention approach

Coalition building through the lens of citizen science was the intervention approach embedded in the larger RADAR project. The research team held the first county-specific coalition planning meetings via zoom in December 2022. We invited citizen scientists and site coordinators to each meeting led by the principal investigator (PI) in which we introduced them to the basics of building local coalitions [17]. Each of the four rural study counties created a systematic plan to form their own community-wide coalition to tackle radon exposure in the home. We empowered each coalition to make their own decisions about specific radon activities that meet the needs of their communities. We developed a RADAR presentation for each site PI and coordinator to promote the inclusion of radon within existing coalitions or to form a new coalition. Site PIs presented to the existing health coalitions in County B and County D (pop. 26 835 and 63 063, respectively). Citizen scientists in attendance offered personal testimony about radon testing and mitigation. These two existing coalitions agreed to add radon risk reduction to their missions, and radon activities began in each county.

As Counties A (pop. 73 995) and C (pop. 23 333) had no active health coalitions at the time, site PIs and coordinators explored coalition building opportunities with community-based organizations such as hospitals and low-income housing groups. County C re-established a formerly active radon coalition and held its inaugural coalition meeting in May 2023. In County A, we approached a recently formed lung health coalition focused on tobacco cessation and lung cancer screening. Given the common goal of improving lung health, coalition leaders agreed to expand outreach activities to include radon risk reduction. County A’s coalition held its inaugural meeting in January 2024.

We used a Culture of Health approach to guide each of the four coalitions in ensuring broad membership across diverse community sectors such as education, housing, public libraries, and healthcare [25]. Coalitions reviewed their membership for both grassroots (e.g. citizen scientists, realtors) and grass-tops organizations (e.g. regional cancer prevention program; housing administrators) and planned to recruit members as appropriate. Types and numbers of members varied across coalitions, with some similarities. As such, the radon coalitions in each of the four counties grew in number and level of engagement over time.

Each of the four coalitions implemented varied radon awareness activities, engaged in different marketing strategies, and actively promoted the radon detector kit LLP in different and unique ways. Some coalitions were focused more on promoting radon testing; others also addressed radon mitigation. Coalition meetings were held regularly in each county emphasizing January as National Radon Action Month (NRAM). Three of the four county coalitions (County B, C, D) invited the County Judge-Executives to sign an NRAM proclamation. NRAM marketing strategies included meeting presentations, direct mail, social media posts, newspaper articles, and tabling at libraries alongside the State Radon Program staff. During the other months, three of the coalitions distributed direct mail postcards to promote the LLP (County B, C, D); County A coalition used paid and unpaid radio spots, including live radio interviews, and engaged with local health care provider offices about radon; County D used paid marketing in the local community magazine to promote use of the LLP. All four coalitions used social media posts, created by the RADAR research team, to raise radon awareness and promote radon testing in their respective communities. Other coalition activities included tabling at the library, wellness fairs, symposiums, and 5 K walk-run events, as well as direct health care provider engagement, live radio interviews, and postings to social media. To promote citizen scientist engagement, RADAR citizen scientists were invited to a private Citizen Scientist Facebook page where they learned of coalition activities and outreach opportunities [24]. Coalition activities reported here were implemented by four different coalitions and evaluated over a two-year period, 2023–2024.

## 4. Methods

We used mixed methods to evaluate coalition-building and citizen science using the RE-AIM framework. There were four data sources for the evaluation: (1) Citizen scientist survey, (2) coalition tracking survey completed by site PIs and research staff, (3) LLP participant survey, and (4) key informant interviews with the four coalition chairpersons. The institution’s Medical Review Board approved the study.

The **Citizen scientist survey** was conducted twice per year. The survey measured study constructs reported elsewhere [22, 23]. We used 14 survey items to measure the citizen scientists’ report of the coalition’s reach, effectiveness, adoption, and implementation (see RE-AIM constructs section below).

The **Library Loan Program (LLP) participant survey** was a one-time post-radon testing set of items asked of homeowners who checked out a radon detector from the library, tested their home, and agreed to participate in the larger study [23]. For this evaluation, we analyzed two items from the LLP survey among those who had high radon levels (*n* = 65) to assess how likely they were to hire a certified radon mitigation specialist and if having financial help to cover the cost would assist in installing a home radon mitigation system. We aggregated the data into seven quarters to assess change over time. To evaluate the radon coalitions, we describe the conceptual and operational definitions of each of the RE-AIM constructs (see table 1).

**Table 1.** RE-AIM construct definitions and measures.

RE-AIM indicator	Conceptual definition	Operational definition	Measures
Reach	Degree of engagement with the target population on the radon coalitions.	Absolute number and representativeness of individuals, groups, and citizen scientists who participated in radon coalitions, as well as community dissemination metrics from coalition activities.	1. RADAR citizen scientist surveys 2. Coalition-reported outreach activities

Effectiveness	Perceived benefits, challenges, and outcomes associated with radon coalitions.	Citizen scientists’ perceptions of cohesiveness and media outcomes associated with the radon coalition.	1. RADAR citizen scientist surveys

Adoption	How well community organizations were engaged in radon coalitions.	Support and resources provided by organizational partners.	1. RADAR citizen scientist surveys

Implementation	The use of best practices and likelihood of hiring a certified radon mitigation professional to fix their home.	Citizen scientists’ and coalition risk reduction activities, including promotion of library loan program.	1. RADAR citizen scientist surveys 2. Library loan participant survey

Maintenance	The perceived facilitators and challenges to coalition sustainability.	Coalition chairperson’s opinions of the facilitators and challenges to coalition sustainability.	1. Key informant interviews with coalition chairperson

**Reach**. We defined how well we reached the target population as the absolute number and representativeness of individuals and citizen scientists who participated in the radon coalitions, as well as dissemination metrics from coalition outreach activities. The data sources for the Reach construct were the citizen scientist survey (3 items) and the coalition tracking survey. First, we asked citizen scientists how involved they were in the coalition on a scale of 1 (not involved at all) to 4 (very involved). Participants could also select ‘I am not aware of a radon coalition in my county.’ If participants reported not involved at all or not aware of the coalition, we coded the response as uninvolved. Second, we assessed the types of organizations that were contacted for partnerships. Third, we measured the frequency of coalition activities to share radon information through various forms of media, advertising, local radon data collection, radon presentations, training healthcare providers, promoting the LLP, and sponsoring community events. The research team also used an internal coalition tracking survey in REDCap to document coalition activities in real time (e.g. meetings, library outreach, and internal/external communications) and update the coalition membership roster. REDCap (Research Electronic Data Capture) is a secure, web-based software platform designed to support data capture for research studies, providing (1) an intuitive interface for validated data capture; (2) audit trails for tracking data manipulation and export procedures; (3) automated export procedures for seamless data downloads to common statistical packages; and (4) procedures for data integration and interoperability with external sources [26, 27].

**Effectiveness**. We defined how effective coalitions were by assessing citizen scientists’ perceptions of benefits, challenges, and outcomes associated with their radon coalition. The data source for the Effectiveness construct was the citizen scientist survey (4 items). First, we assessed effectiveness in resolving conflicts among coalition members on a scale of 1 (not at all effective) to 4 (very effective), with higher scores meaning effective conflict resolution. Second, we measured their view of the coalitions’ climate on a scale of 1 (not at all cohesive) to 4 (very cohesive) with higher scores reflecting a more positive climate. Third, we assessed the frequency of media attention toward radon by type (e.g. newspaper, radio, social media) on a scale of 1 (none) to 5 (frequent), with higher scores reflecting more media attention toward radon awareness, testing or mitigation in the past 6 months. Lastly, we evaluated the favorability with how radon was portrayed by diverse types of media on a scale of 1 (unfavorable) to 5 (favorable), with higher scores meaning greater favorability.

**Adoption**. We defined how well community organizations were engaged by assessing the levels and intensity of organizational and community radon coalition participation. The data source for Adoption construct was the citizen scientist survey (4 items). The survey items were focused on testing and mitigation of existing homes, rather than new construction. First, we asked what types of housing-affiliated groups oppose radon awareness, testing, or mitigation (e.g. realtors, home inspectors, insurance companies, banks, other). Since Kentucky’s building code does not mandate the use of radon resistant new construction techniques, we did not specifically include builders in the response options, however citizen scientists could specify them by selecting ‘other.’ Second, we asked if the coalition had conducted events to educate opposition groups (yes/no). Third, we assessed how supportive partnership organizations were for radon efforts on a scale of 1 (not supportive at all) to 5 (very supportive), with higher scores meaning more support. Lastly, we asked citizen scientists to rate the level of resources (e.g. financial, space) provided by the partnership organizations on a scale of 1 (none) to 5 (extensive resources), with higher scores reflecting more resources.

**Implementation**. We defined implementation as the use of best practices and the likelihood of hiring a certified radon mitigation professional to fix their home. The data sources for the Implementation construct were the citizen scientist survey (1 item) and the library loan participant survey (2 items). First, we assessed the citizen scientists’ experiences with educating the public through a variety of best practices [28] (7 response options) on a scale of none [1] to extensive [5]. To evaluate the impact of the coalitions on radon mitigation, we evaluated whether residents who checked out a radon detector from the library and found high levels of radon greater than or equal to 4.0 pCi l^−1^ (148 Bq m^−3^) were likely to mitigate for radon. Of those with a radon value at 4.0 pCi l^−1^ or higher, we asked about hiring a certified radon mitigation professional to fix their home on a scale of 0 (very unlikely) to 4 (very likely). For the analysis we dichotomized the responses as very unlikely/unlikely or somewhat likely/very likely. We also assessed whether having financial help to pay for radon mitigation would assist them in installing a mitigation system (yes/no).

**Maintenance**. We defined the facilitators and challenges to coalition sustainability using qualitative methods. We sent an email invitation to each coalition chairperson at the end of the study to take part in a 60 min virtual key informant interview. A member of the research team used a semi-structured interview guide to facilitate the discussion, and a project assistant recorded field notes to summarize key points and nonverbal responses. The interview guide consisted of 8 open-ended questions and prompts to assess which aspects of the coalition were most effective and most challenging, as well as which approaches were perceived as most helpful or difficult in supporting coalition growth. Participants were also asked to describe concerns regarding their county’s ability to sustain the coalition. We recorded and transcribed each interview using the zoom online meeting platform. Using content analysis, three authors [EH, NC, SS] coded the data to identify categories and themes related to facilitators and challenges of coalition sustainability.

## 5. Results

**Reach**. The County D existing coalition comprised the most members (*n* = 34), followed by County B with 32 members, and County A (new health coalition) with 18 coalition members. These three coalitions focused on health and wellness more broadly. County C re-formed a radon-only coalition and had the fewest members (*n* = 10). Across the 4 counties, coalition members represented the public library, hospitals, healthcare clinics, cancer prevention and control programs, area health education centers, health departments, housing, local government, libraries, schools, businesses (e.g. insurance companies; real estate brokerage), parent groups, citizen scientists, and organizations serving low-income populations such as affordable housing and community action councils.

A total of 44 citizen scientists (*N =*46; 96% participation rate) completed the citizen scientist survey at end of study. Slightly less than half (41%) were at least moderately involved in the coalition. From a list of 15 organizations, the most frequently reported organizations contacted for possible partnerships for radon efforts included local health departments (22%), local hospitals (11%), and schools (11%). Among those who reported coalition involvement (*n* = 18), the most frequently reported coalition activities included distributing information about the LLP (86%) and setting up a display or table at the library to promote the program (79%), collecting local data on radon (71%), distributing pamphlets on radon (69%), and posting social media ads about radon (57%). There were no significant differences in demographic characteristics, family history of lung cancer or having high radon in their home between those who were and were not involved in the coalition (see table 2).

**Table 2.** Sociodemographic characteristics of citizen scientists by coalition involvement.

	Any coalition involvement (*n* = 18)	Not involved (*n* = 26)	*p*
Age	54.3 (15.0)	47.5 (12.8)	.12

Sex			.65
Male	6 (33.3%)	7 (26.9%)	
Female	12 (66.7%)	19 (73.1%)	

Race/ethnicity			.40
White, non-Hispanic	18 (100.0%)	25 (96.2%)	
Hispanic or other	0 (0.0%)	1 (3.8%)	

Education			.36
High school	15 (83.3%)	24 (92.3%)	
Beyond high school	3 (16.7%)	2 (7.7%)	

Family history of lung cancer			.91
Yes	3 (16.7%)	4 (15.4%)	
No	15 (83.3%)	22 (84.6%)	

High radon in the home			.55
Yes	6 (33.3%)	11 (42.3%)	
No	12 (66.7%)	15 (57.7%)	

**Effectiveness.** Among those involved in the coalition, over half said their coalition was effective or very effective (51%) in constructively resolving conflicts among members, and the current climate among members was cohesive or very cohesive (53%).

For all media types, citizen scientists’ awareness of media attention and favorability of radon awareness, testing and mitigation was low, particularly among those uninvolved in the coalition (see table 3). The percentage of participants who responded ‘do not know’ to attention in the media was significantly higher among those not involved compared to those involved in the coalition for all types of media. Similarly, the uninvolved citizen scientists were more likely to respond ‘do not know’ related to favorability of how radon was portrayed by all but two types of media (local newsletters and cable television). Among those who indicated a response other than ‘do not know’ to media attention inside the library, there was a significant difference by coalition involvement, with coalition-involved citizen scientists rating higher than those who were non-involved (*M* = 4.8, *SD* = 0.4 vs. *M* = 4.2, *SD* = 1.1; *p* = .026).

**Table 3.** Attention and favorability toward radon awareness, testing, or mitigation by type of media (*N* = 44).

	Attention				*p* ^a^	Favorability				*p* ^a^
	Involved		Not involved			Involved		Not involved		
	mean (*SD*)	% Don’t know	mean (*SD*)	% Don’t know		mean (*SD*)	% Don’t know	mean (*SD*)	% Don’t know	
Newspaper	2.4 (1.4)	31%	3.0 (1.6)	72%	.010	3.5 (0.9)	31%	4.0 (1.0)	84%	<.001
Radio	2.8 (1.0)	27%	2.1 (1.1)	63%	.029	3.5 (0.9)	40%	4.0 (1.0)	75%	.029
Billboards	2.5 (1.6)	7%	2.0 (1.0)	54%	.003	3.0 (1.4)	33%	5.0 (n/a)	74%	.013
Social media	3.5 (1.3)	7%	3.1 (1.5)	36%	.038	3.8 (0.9)	13%	4.2 (1.0)	65%	.002
Local newsletters	2.6 (1.3)	40%	2.3 (1.2)	75%	.029	3.0 (1.6)	53%	5.0 (0.0)	74%	.19
Cable television	1.7 (0.8)	33%	1.8 (1.8)	67%	.042	1.7 (0.6)	60%	—	83%	.10
Inside the public library	4.8 (0.4)	0%	4.2 (1.1)	28%	.024	4.6 (0.6)	0%	4.2 (0.9)	42%	.004
Websites	3.6 (1.4)	7%	3.1 (1.4)	50%	.007	3.9 (1.1)	14%	4.6 (0.5)	71%	<.001

*Note:* Response option ranged from 1) none to 5) frequent attention/favorability toward radon awareness, testing, or mitigation on types of media in the past 6 months.

^a^ *p* from chi-square test of association comparing percent do not know responses by coalition involvement.

One important Effectiveness outcome from the public education and coalition work in County C was a new Housing and Urban Development (HUD) Healthy Homes Production grant to the local affordable housing organization to provide health and safety remediation in homes including radon testing (referral to the LLP) and mitigation for up to 100 homes in the region.

**Adoption**. When asked to select which housing-affiliated groups (e.g. realtors; home inspection companies; insurance companies; banks) currently oppose radon awareness, testing and or mitigation in the county, the majority responded, ‘do not know’ (88%); 10% selected realtors, 2% chose all four groups, and 2% wrote in landlords. There was no difference in the rate of ‘do not know’ responses by coalition involvement. Of those involved in the coalition, 16% said their coalition had educated groups who oppose radon awareness, testing or mitigation.

When asked to consider the level of support received from the most frequently contacted organizations, citizen scientists identified local health departments, local hospitals, and schools as being highly supportive (*M* = 4.5, *SD* = 0.7, *M*= 5.0, *SD* = 0.0 and *M* = 4.5, *SD* = 0.70, respectively); yet they provided few to no resources to the coalition. However, using a mixed methods approach allowed us to discover an unexpected outcome, as the County D coalition provided financial support to two homeowners covering their entire radon mitigation expenses totaling $2852.

**Implementation**. Citizen scientists reported moderate levels of experience with seven different coalition activities (e.g. organizing grassroots supporters, fundraising). They had the most experience disseminating scientific information on radon *(M* = 3.8, *SD* = 0.8), educating the public to raise radon awareness *(M* = 3.7, *SD* = 0.8; see table 4), and conducting media campaigns *(M* = 3.2, *SD* = 1.3). The percentage of citizen scientist coalition members who responded ‘do not know’ ranged from 47% to 72%. There were no differences based on involvement in the coalition.

**Table 4.** Coalition member experience with different coalition activities.

	Experience mean (*SD*)	% Do not know
Organizing grassroots supporters	3.1 (0.9)	62%
Educating the public to raise radon awareness	3.7 (0.8)	49%
Disseminating scientific information on radon	3.8 (0.8)	50%
Conducting media campaigns	3.2 (1.3)	47%
Fundraising	2.2 (1.3)	66%
Working with low-interest loan providers	2.0 (1.3)	72%
Working with radon mitigation companies	2.3 (1.3)	63%

*Note*: Response option ranged from (1) none to (5) extensive.

**Figure 1.**
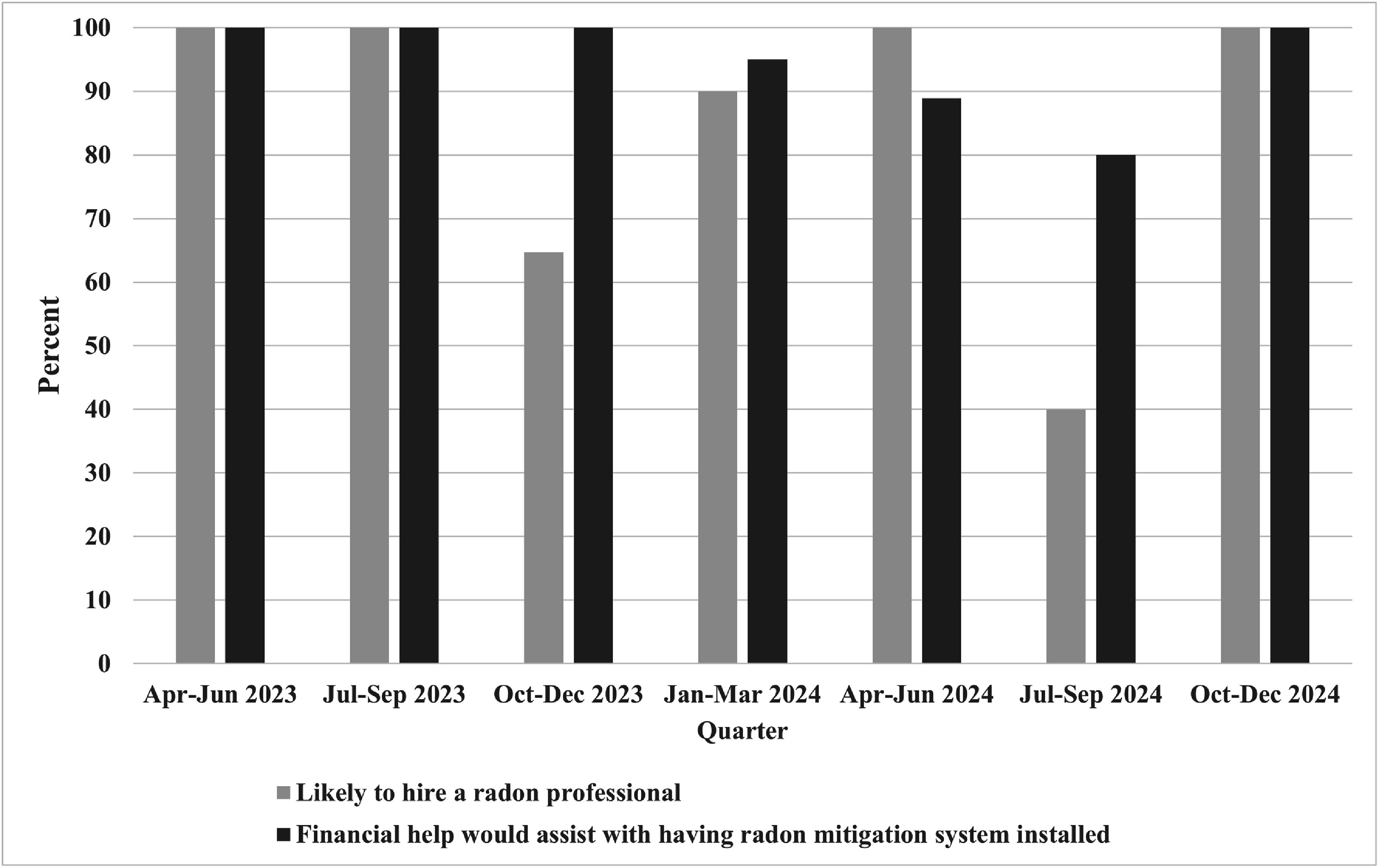
Likelihood of library loan participants to hire a radon mitigation professional and financial motivation to assist with mitigation across the study period.

Of the 65 LLP participants who reported having a high radon value on any of the days they tested, the number of responses per quarter ranged from 2 (Apr–Jun 2023) to 20 (Jan–Mar 2024). The percentage of participants who said they were likely to hire a radon mitigation professional ranged from 40% to 100% each quarter, yielding a total of 82% across the study period. Nearly all (95%) said that financial assistance would assist them to mitigate for radon (see figure 1). Due to the limited number of responses per quarter, we were unable to evaluate changes over time with repeated measures modeling, and we summarized the data descriptively.

**Maintenance**. All four chairpersons participated in the key informant interviews. Three were female and one was male. All were paid staff at their respective workplaces (e.g. health department [2], area health education center, cancer prevention and control program).

Four key facilitators of radon coalition sustainability were identified. First, multi-issue health coalitions that included radon into their mission had greater member engagement and less concern about sustainability. By incorporating radon into the mission of the multi-issue health coalitions, it prompted home radon testing among members, which in turn supported radon outreach with different community groups as members were more willing to share their lived experience with testing a home for radon. Furthermore, by addressing a variety of community health concerns in the multi-issue health coalitions, members were able to leverage their network, share resources, and take collective action toward common goals that promote community health:

‘I think one of the main benefits is that we can all network and talk about all the resources we have and help each other out because we are all working towards the same goal, so I honestly foresee this continuing.’

Second, involvement from influential community organizations (e.g. local government, health departments, schools) promoted coalition credibility by providing name recognition and increasing public attention to coalition activities. Chairpersons described how residents were more likely to take radon seriously and take action to test their own home if these key organizations were part of the coalition:

‘….the AHEC [Area Health Education Center] was great at getting the education out there… and the County Judge-Executive getting things [National Radon Action Month proclamation] signed, to say that we are going to do this together as a county.’

Third, coalitions with stable funding (e.g. from grants, fundraising) expressed more confidence in sustainability. Having financial support enabled the coalitions’ ability to offer meeting incentives (e.g. light refreshments), host and participate in community health events, distribute radon educational materials, utilize targeted marketing for the LLP (e.g. community magazine), and offer mitigation assistance.

‘We were able to take a homepage out in a magazine that goes to 30 000–40 000 community leaders …. We took those out a couple times… we were able to get some pens and different things made where we could advertise and get it [radon library loan program] out there.’

Lastly, varied coalition approaches to public education fostered radon awareness and action among community members. Coalition efforts included distribution of health-focused radon materials; proclamations by government officials; social media outreach; flyer distribution through community partners (e.g. food pantries; schools); media campaigns; and event participation. These coalition efforts expanded reach, increased community knowledge, and promoted the LLP and home radon testing. Presence at community health events, as well as radio and mailed promotions, helped reach people who did not often go to the library. Chairpersons perceived knowledge about radon had improved over time, and the more people knew about radon, the more likely they were to test their home and tell others about their experience, promoting sustainability by increasing community buy-in and engagement.

In contrast, we identified three key challenges of radon coalition sustainability. First, coalitions whose mission was limited to radon-only experienced lower member engagement and greater sustainability concerns as competing community health priorities (e.g. substance use) limited participation and perceived relevance. Multi-issue health coalitions were viewed as more viable long-term:

‘…. We have come through the pandemic, and … the aftermath …… substance use is top of mind for so many folks here…. the radon issue [alone] just hasn’t seemed to get anybody energized to attend meetings. ‘It [the coalition] has a much higher likelihood of sustaining long-term…if it is part of something bigger rather than just a radon coalition.’

Second, low coalition commitment from residents and organizations presented challenges with maintaining momentum. Seasonal gaps in meetings (i.e. no summer meetings), limited citizen scientist involvement, and in some cases, inconsistent engagement from public libraries due to turnover in leadership and competing priorities, presented challenges with keeping radon on the coalition’s agenda. As the public libraries housed the LLP and, in some cases, served as meeting venues for the coalitions, limited library engagement may have reduced coalition outreach capacity and contributed to missed opportunities for promoting home radon testing:

‘we have had 3 library directors, and looking for a fourth. A lot of staff turnover, so I just think we have had a lot of challenges that have minimized the impact that we perhaps could have had.’

Lastly, lack of access to radon professionals undermined radon action; residents reported long wait times when working with radon measurement and mitigation companies and the need to reach outside local areas to hire a radon company led to discouragement and frustration, weakening participation in home radon testing and recommended follow-up efforts:

‘…when we called them, it was going to be 6 months to a year before they could do it…we had to go all the way to [a large urban area] to find a radon mitigation professional.’

## 6. Discussion

We implemented health coalitions to address radon, a persistent public health challenge that warrants community-wide collaboration. Our findings confirm coalition building is a powerful public health tool that can build community social capital, focus on a common goal, and attract influential key partners as well as community activists [4, 14]. However, few coalitions address this important public health challenge [15, 16]. Making radon testing more accessible by partnering with public libraries [24] and health coalitions to raise radon awareness, testing, and mitigation shows promise. Three of the four rural study counties had multi-issue, multi-partner health coalitions that addressed radon among other community health concerns. The multi-issue, multi-stakeholder coalition is the most enduring form of local coalitions [4]. Only one coalition in our study specifically focused on radon; it was the smallest with only 10 members. Our study revealed concerns with single-focused local coalitions and suggests that, in communities facing more pressing health concerns (e.g. substance use), single-issue coalitions may struggle to attract and sustain participation. In contrast, Bain *et al* describe a successful state-level, single-focused radon coalition comprised of local and state public health departments, non-profit health groups, lung cancer survivors and others [14]. The primary local coalition partners in our study were from fields traditionally expected in public health (e.g. health departments, hospitals, schools). Future coalition-building interventions might consider reaching non-traditional coalition partners such as realtors and homeowner associations as potential coalition members. However, our findings showed that realtors may be perceived as opponents to radon risk reduction efforts. Perhaps, this finding was due to lack of widespread outreach and education with realtors in the study counties. Momin *et al* report that realtor attitudes and concerns toward radon testing and mitigation vary; some expressing skepticism while others viewed radon testing during home sales as more of a financial issue than one of health [29]. With more outreach and education with realtors, they may be effective allies to tackle this intractable public health challenge.

The coalition partners were very supportive, but they provided few to no financial resources for the coalition. This is a common challenge as members and organizations in rural, socioeconomically disadvantaged regions often have scarce financial resources [4]. In this study, we found stable funding facilitated coalition sustainability, supporting member engagement and coalition outreach activities; as was the case with County D’s coalition which was able to provide mitigation support for several households. While the other three coalitions may have lacked the funding or the focus on radon mitigation to support their community in this way, it was encouraging that one coalition partner in the affordable housing sector was successful in leveraging a federal grant to promote radon testing and financial support for radon mitigation. Further, the fact that one coalition provided mitigation support for several households shows the importance of not only evaluating partner support but also assessing resources they provide, using mixed methods.

Our evaluation of LLP patrons who checked out free radon detectors showed that nearly all with high radon reported they would hire a radon mitigation professional to fix high radon. This finding is consistent with the Iowa experience, showing dramatic increases in demand for radon mitigation after implementation of their state radon coalition [14]. In our study, variability in the likelihood of hiring a radon mitigation professional over time may have been due to coalition factors such as radon-focused community events in certain months (e.g. NRAM in January) and fewer coalition meetings in the summer months. We need further evaluation and research to determine the role of health coalitions in promoting remediation of health challenges.

One strength of our coalition approach was that we engaged citizen scientists who brought a valuable skill set to the coalitions. The fact that disseminating scientific information about radon was the most reported coalition activity reveals the power of the citizen science approach [30]. Citizen scientists were trained in all aspects of evidence-based radon risk reduction [22], and they were confident in their ability to disseminate scientific information on radon. Further, citizen scientists who participated in the coalitions were more attentive to general media coverage related to radon awareness, testing, and mitigation and viewed media coverage as more favorable compared to those uninvolved in the coalition. Further, these citizen scientists were more aware of the media attention inside the libraries than those not involved in the coalition. However, engagement in coalitions is labor-intensive [4] and this could be why only four in 10 citizen scientists were involved in the coalitions. Lastly, the fact that the citizen scientists had tested for radon and were an integral part of the radon detector LLP may have been one reason why the library loan patrons with high radon were likely to hire a certified radon mitigation professional.

The fact that the coalitions were new to radon risk reduction is a limitation of this evaluation. In addition, we interviewed only coalition chairpersons to assess the Maintenance construct; coalition sustainability perspectives from other coalition members and citizen scientists were not captured. Thus, our findings primarily reflect leadership viewpoints and may not fully represent the experiences or perceptions of all coalition participants. Furthermore, we partially relied on citizen scientists’ self-report to evaluate the coalitions, and many did not know the answers to our questions. This may have been due to the limited intensity of coalition involvement or the fact that the coalition’s focus on radon was a new goal. Coalition-building for radon risk reduction was in the early formation phase [17] in the four rural communities. We may have observed fewer ‘do not know’ responses in later phases of the coalitions, as members would have been more involved and informed over longer periods of time. However, a strength of our evaluation is that most coalitions built on multi-issue health efforts to promote sustainability over time. Replication of this evaluation would shed more light on the impact of citizen scientist-engaged health coalitions on radon awareness, testing, and mitigation in rural communities.

## 7. Conclusion

In summary, coalition building represents a powerful public health strategy for uniting stakeholders around shared goals and engaging both influential partners and grassroots community members to advance radon awareness, increase home radon testing, and build demand for radon mitigation in rural communities. Findings suggest that multi-issue coalitions that integrate radon within a broader health agenda and are able to maintain stable funding demonstrate greater reach, effectiveness, and sustainability than radon-only efforts. These coalitions are well positioned to achieve meaningful and lasting community impact by leveraging diverse partnerships, sustained engagement, and shared resources to address radon risk alongside other community health priorities. Support from citizen scientists and community partners engaged in radon awareness efforts helped the issue stay on the agenda and facilitate change.

## Data Availability

The data cannot be made publicly available upon publication because the cost of preparing, depositing and hosting the data would be prohibitive within the terms of this research project. The data that support the findings of this study are available upon reasonable request from the authors.
